# Iodine deficiency in pregnant women after the adoption of the new provincial standard for salt iodization in Zhejiang Province, China

**DOI:** 10.1186/s12884-018-1952-5

**Published:** 2018-08-03

**Authors:** Guangming Mao, Wenming Zhu, Zhe Mo, Yuanyang Wang, Xiaofeng Wang, Xiaoming Lou, Zhifang Wang

**Affiliations:** 1 Department of Environmental and Occupational Health, Zhejiang Provincial Center for Disease Control and Prevention, Hangzhou Zhejiang, Province, People’s Republic of China; 2 Key subject for medical research, Zhejiang Provincial Center for Disease Control and Prevention, Hangzhou Zhejiang, Province, People’s Republic of China

**Keywords:** Iodine, Iodine deficiency, Urinary iodine concentration, Pregnancy

## Abstract

**Background:**

Zhejiang has achieved the goal of elimination of iodine deficiency disorders (IDD) via the implementation of universal salt iodization (USI) since 2011. Iodine content in household table salt decreased from the national standard (35 ppm) to the Zhejiang provincial standard (25 ppm) in 2012. It is crucial to periodically monitor iodine status in pregnant women because IDD in pregnancy have adverse effects on fetal neurodevelopment.

**Methods:**

We carried out a cross-sectional study between April 2014 and September 2015 in the eight sentinel surveillance counties across Zhejiang Province, where IDD was previously known to be endemic. A total of 1304 pregnant women participated and provided a random spot urine sample and a household table salt sample. Urinary iodine concentration (UIC) was determined using arsenic-cerium catalytic spectrophotometry. Iodine content in salt was measured using a titration method with sodium thiosulphate.

**Results:**

Overall, the median UIC of the total study population of pregnant women was 129.3 μg/L, with a higher UIC in inland (152.54 μg/L) and a lower UIC in coastal counties (107.54 μg/L). Household coverage of iodized salt was 94.6% and the rate of adequately iodized salt was 89.9%.

**Conclusions:**

Our results indicate deficient iodine status in the pregnant population of Zhejiang, according to the lower cut-off value of optimal iodine nutrition (150 μg/L) recommended by the World Health Organization. In addition to sustaining USI, more efforts are urgently needed to improve iodine intake in women during pregnancy, especially those residing in the coastal counties.

## Background

Iodine is an essential micronutrient in human beings for healthy brain development. Inadequate iodine intake cause iodine deficiency disorders (IDD), which affects humans at all stages of the life cycle, especially in pregnancy. IDD in pregnancy damages the neurodevelopment of the fetus and can lead to stillbirth, miscarriage, mental retardation, congenital abnormalities, dwarfism, hearing loss, and other problems [1]. Hence, maintaining adequate iodine intake is crucial for pregnant women.

The iodine requirement increases during pregnancy due to physiological changes in iodine metabolism [2], including increased renal clearance of iodide as well as the additional iodine requirement of the fetus and of the mother to synthesize thyroid hormone, to maintain euthyroidism. According to the World Health Organization (WHO) recommended assessment of iodine nutritional status, pregnant women have a higher iodine requirement (250 μg/day/person) than the general population (150 μg/day/person). There is an approximately 50% increase of thyroid hormone production during pregnancy, which puts pregnant women at high risk of iodine deficiency.

Zhejiang Province, located in Eastern China, has been proved to be a region where the iodine content in the environment (e.g. soil and drinking water) is too low to maintain optimal iodine status in population [3]. Iodine deficiency was documented as a severe public health problem in Zhejiang as early as the 1970s [4]. In 1984, epidemiological studies in the province showed that 532,020 schoolchildren aged 7–14 years (32.6%) showed visible signs of goiter, and approximately 12 million people were estimated to be at risk of IDD, accounting for 3% of the total population of China [5]. Iodized salt, containing at least 20 ppm at the household level, was distributed only in towns where the prevalence of goiter among schoolchildren was 30–70% between 1984 and 1986 [6]. By 1995, more than 97.2% of towns had been intervened with iodized salt (35 ppm; range: 20–50 ppm) and universal salt iodization (USI) then had been introduced throughout the whole province. Zhejiang achieved the goal of IDD elimination in 2011 based on the indicators monitoring in school-age children, which are taken as a good proxy for the general population [7].

The results of Chinese national surveillance of IDD showed that the median urinary iodine concentration (UIC) in school-age children in Zhejiang Province was 237 μg/L in 2011 [8], denoting more than adequate iodine intake in the general population according to the WHO recommended lower cut-off value of 200 μg/L. This result indicated that the susceptible population groups may be at risk of iodine-induced hyperthyroidism. Gradually increased incidence of thyroid disorders was reported after the implementation of the USI programme, for which complaints were received [9]. Since iodized salt is the most significant single source of iodine intake in China, the iodine content in salt (35 ppm; range 20–50 ppm) was presumed to be set higher, causing more than adequate iodine intake in the population. Therefore, the Zhejiang Provincial government was authorized to choose a lower standard for salt iodization in an attempt to minimize the incidence of hyperthyroidism related to consumption of iodized salt. A new provincial standard for iodized salt (25 ppm; range:18–33 ppm) was then adopted in 2012.

It is necessary to regularly monitor the iodine status in the population, especially in the most vulnerable population such as pregnant women. The aim of this study was to assess iodine nutritional status among pregnant women in Zhejiang Province, after this new standard of iodized salt (25 ppm) has been implemented.

## Data and Methods

### Study design and sample collection

Participants were recruited from the eight sentinel surveillance counties across Zhejiang (Deqing, Changxing, Wencheng, Yunhe, Panan, Daishan, Shengsi, and Yongjia county) between April 2014 and September 2015. The included counties are remote, impoverished areas where endemic cretinism was previously known to be endemic or household coverage of iodized salt was lower than 80%. Sampling was in accord with the 2014–2015 Chinese National IDD Surveillance guidelines [10]. In each sentinel surveillance county, five towns were randomly selected from five different geological locations (east, west, north, south and center). In each selected town, 30 pregnant women attending antenatal care in township health centers were invited to participate in this study. Healthy participants residing in the selected town for at least 6 months were included. Participants self-reporting a history of thyroid disease and chronic medication were excluded. For each participant enrolled, approximately 20 mL of a random spot urine sample was collected and sealed in a polypropylene tube with a screw top. Each participant also provided approximately 50 g of household table salt, which was collected in a plastic bag. Spot urine samples were immediately refrigerated at 4 °C. Household table salt samples were kept in a dark place at room temperature. Each participant’s birthdate and postcode were recorded.

The iodine content in tap water at each sampling site in each selected county was extracted from the 2011 Chinese National IDD Surveillance (CNIS 2011). The CNIS 2011 was conducted by the Chinese Center for Disease Control and Prevention (CDC). The CNIS 2011 was intended to provide data for decisions regarding the provision of iodized salt, according to the iodine content of drinking water in regions throughout the country.

### Determination of iodine content

Iodine content in salt was measured using a titration method with sodium thiosulphate (GB/T 13025.7–2012). Precision was less than 2 ppm. UIC was determined using arsenic-cerium catalytic spectrophotometry (WS/T 107.1–2016). In a 2.5 mL urine sample, the limit of detection was 2.0 μg/L. The recovery of added iodine was 98.6%.

All the iodine testing laboratories participated in an interior quality control and an external quality assurance programme run by the Chinese CDC. Iodine levels of all samples were examined in the Chinese National Reference Laboratories. According to the assessment criteria recommended by the WHO [11, 12], iodine nutritional status in pregnant women are deficient when the median UIC is less than 150 μg/L while iodine status is sufficient when the median UIC remains between 150 and 249 μg/L.

### Statistical analysis

Data were input in Microsoft Office Excel 2007 and analyzed using IBM SPSS version 23.0 (IBM Corp. Armonk, NY, USA). The Kolmogorov–Smirnov test was performed for normality. Non-normally distributed UICs were expressed as median and interquartile range (IQR). The spread in UIC was described as the frequency. The median UICs between two or more groups were compared using non-parametric Mann–Whitney or Kruskal–Wallis tests. A non-parametric Spearman correlation test was used to assess the relation between environmental iodine contents (e.g. salt and drinking water) and UIC. Binary logistic regression analysis was performed to investigate the association between geographical location (the coast or inland) and category of household table salt (iodized or non-iodized salt) to establish the probability of iodine deficiency. *P*-values <0.05 were considered significant.

According to iodine ion content determined in salt, household table salt samples were classified into four groups: non-iodized (< 5 ppm), inadequately iodized (5–17.9 ppm), adequately iodized (18–33 ppm), and excessively iodized (> 33 ppm). Based on the distribution of geographical locations, the eight selected counties were categorized into the coastal region, including Wencheng, Daishan, Shengsi and Yongjia county; and the inland including Deqing, Changxing, Yunhe, and Panan county.

## Results

A total of 1370 pregnant women in Zhejiang Province were selected to participate in this study. Of the total study population, 95.2% (1304) of participants provided both a urine sample and a household table salt sample. The mean age of the pregnant women was 28.0 years, with a standard deviation of 4.5 years. A total of 109 samples of drinking water were obtained for iodine content, to establish the association between iodine concentration in drinking water and urinary iodine excretion among participants in the selected regions.

A Kolmogorov–Smirnov’s test (*P* < 0.001) and visual inspection of its histogram and a normal Q-Q plot showed that the UIC in all participants was non-normally distributed, with a skewness of 2.719 (standard error 0.068) and a kurtosis of 13.447 (standard error 0.135).

**Fig. 1:**
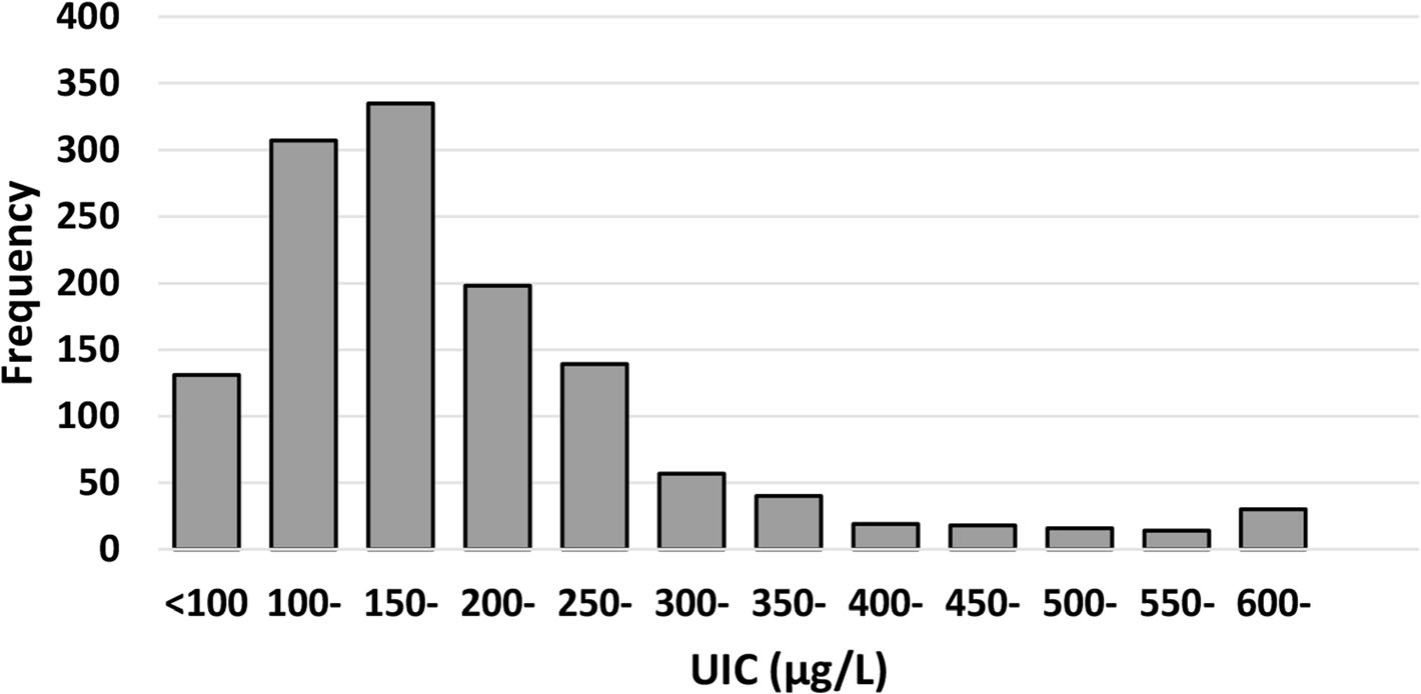
Distribution of UIC in 1304 Pregnant Women in Zhejiang

Overall, the median UIC among the total study population of pregnant women was 129.34 μg/L (IQR: 83.05–201.67 μg/L). Figure 1 showed the frequency distribution of UIC in pregnant women in the eight sentinel surveillance counties across Zhejiang in 2015. Among 1304 pregnant participants, 59.3% (773) had UIC < 150 μg/L and 25.8% (337) had UIC 150–249 μg/L. Among 773 samples with a UIC lower than the WHO recommended lower cut-off level of optimal iodine status (150 μg/L), 56.7% (438) of samples had UIC below 100 μg/L and 43.3% (335) had UIC 100–149 μg/L.

Table 1 shows the median UICs of the total study population of pregnant women distributed by maternal age, category of household table salt, and region. For the total population, participants consuming iodized salt had a significantly higher median UIC (133.13 μg/L) than participants using non-iodized salt, who had the median UIC 96.95 μg/L (*P* = 0.001). The median UIC (152.54 μg/L) of participants residing in the inland areas was significantly greater than the median UIC (107.54 μg/L) of those living in coastal areas (*P* < 0.001). The median UIC of pregnant women was not statistically significantly different among age groups (*P* = 0.973).

**Table 1:** UIC according to maternal age, category of table salt, and region

N (%)		Median UIC(IQR), μg/L	*P*
Maternal age (years old)			0.945^a^,0.986^b^
≤19	13 (1.0)	147.02 (84.06–250.88)	
20–24	214 (16.4)	127.50 (79.65–209.44)	
25–29	635 (48.7)	129.66 (85.23–192.10)	
30–34	303 (23.2)	129.00 (82.28–200.68)	
≥35	139 (10.7)	136.43 (80.10–219.00)	
Category of salt (ppm)			< 0.001^a^, < 0.001^b^
Non-iodized (< 5)	70 (5.4)	84.83 (62.05–105.23)	
Inadequately iodized (5–17.9)	58 (4.4)	107.00 (67.54–190.00)	
Adequately iodized (18–33)	1172 (89.9)	135.00 (94.55–214.00)	
Excessively iodized (> 33)	4 (0.3)	267.00 (88.23–446.00)	
Region			< 0.001^a^, < 0.001^b^
Coast	594 (45.6)	107.54 (75.55–157.83)	
Inland	710 (54.4)	152.54 (97.10–236.63)	
Total	1304 (100)	129.34 (83.05–201.67)	

^a^Mann-Whitney test and Kruskal-Wallis test for two or more groups

^b^Spearman correlation

Of 1304 salt samples, 1234 (94.6%) were determined to be iodized salt and 1172 (89.9%) were adequately iodized salt. Household coverage of iodized salt in the coastal areas (79.8%) was significantly lower than that in the inland counties (96.2%, *P* < 0.001). For participants living in the coastal areas, the median UIC was significantly different between participants who consumed iodized salt (113.78 μg/L) and those using non-iodized salt (88.61 μg/L). For participants living in the inland counties, those who consumed non-iodized salt had a lower median UIC (84.95 μg/L) than those consuming iodized salt (150.50 μg/L). There was a positive association between iodine content in salt and the median UIC (*P* < 0.000). The overall median iodine concentrations in drinking water was 2.28 μg/L (IQR: 0.30–12.65 μg/L). Iodine concentration in drinking water, distributed by region, showed no significant difference (*P* = 0.773), with 5.45 μg/L in the coastal counties and 2.27 μg/L in the inland counties. No association between iodine concentration in drinking water and urinary iodine excretion was identified (*P* = 0.456).

We further established the effects of the category of household table salt (intake of iodized salt or non-iodized salt) and geographical locations (coast or inland) on UICs through logistic regression analysis. The probability of iodine deficiency in the coastal counties was 2.78 times higher than that in the inland ones (*P* < 0.001), and the probability of iodine deficiency among participants using non-iodized salt was 4.72 times higher than that in participants consuming iodized salt (*P* < 0.001; Table 2).

**Table 2:** Probability of iodine deficiency following logistic regression analysis

Variables	B	SE	*P*	OR (95% CI)
Geological location (Coast = 1; inland = 0)	1.022	0.181	0.000	2.78 (1.95–3.95)
Category of table salt (non-iodized = 1; iodized = 0)	1.551	0.42	0.000	4.72 (2.07–10.76)
Constant	0.745	0.144	0.000	2.106

SE: Standard error

## Discussion

Through the implementation of USI programme, China has achieved the goal of elimination of IDD at a national level since 2011 [7]. However, there were regional differences in iodine intake status throughout the whole country. Adequate iodine intake appeared in the coastal regions of China, above requirement iodine intake in the inland regions and excessive intake in parts of the central ones [7, 8]. To further achieve the goal of IDD elimination at a provincial level, in 2012 China authorized 31 provinces to choose their own standard for salt iodization. This means that the one national standard for salt iodization (35 ppm, range: 20–50 ppm) has been changed to different provincial standards ranging from 20 to 30 ppm (GB 26878–2011).

Zhejiang has been consistently believed to be one province that has achieved the goal of elimination of IDD among the general population [13–18]. Our study showed the median UIC among participating pregnant women in Zhejiang was 129.34 μg/L, indicating that these women in pregnancy were iodine deficient. There are no cut-off values for distinguishing among mild, moderate, and severe iodine deficiency during pregnancy, according to the WHO recommendations. Based on the published literature [19–23], the degree of iodine deficiency during pregnancy in this study is defined as mild. Several longitudinal studies have described that marginal or mild iodine deficiency in uterus also have negative effects on fetal brain development [24–29]. We believe that these results are potentially important to public health since mild IDD in pregnant women can have adverse effects on the fetus.

Compared with previously sufficient iodine intake during pregnant women, reported in epidemiological studies conducted in Zhejiang before adoption of the new provincial standard of iodine content in salt [8, 30], our present results confirm that iodine deficiency among pregnant women in Zhejiang has re-emerged. These results are in line with those of other studies conducted in China [31–33]. Chen et al. and Wang et al. observed re-emerging iodine deficiency during pregnancy after the new standard was implemented in Fujian, one of the coastal provinces, where 25 ppm iodine in salt was also adopted [32, 33]. A decreased UIC in pregnant women from 224.9 μg/L in 2011 to 202.5 in 2014 was observed in Henan where the provincial standard for salt iodization was decreased to 30 ppm after 2012 [34]. Similarly, the UIC in school-age children in Henan dropped from 315 μg/L in 2005 to 204 in 2014 [34]. In addition, we observed that 94.6% of pregnant women consumed iodized salt and 89.9% used adequately iodized salt, which is close to the goals of IDD elimination for coverage rate of iodized salt at the household level (≥95%) and for rate of adequately iodized salt (> 90%) according to the Chinese National Standard of IDD Elimination (GB 16006–2008). However, those consuming iodized salt were still mildly iodine deficient. Taken together, we can speculate that inadequate iodine intake re-emerging in pregnant women may be related to the new provincial standard of decreased iodine content in household table salt. We therefore suggested that it is necessary to choose a higher iodine content in salt (30 ppm, range: 21–39 ppm) for pregnant women that is still within the Chinese national permitted range. Further researches are needed to elucidate this content. More efforts should be made to increase iodine intake among pregnant women, in addition to implementing the USI strategy. For example, a recommendation could be made for pregnant women to take iodine-containing supplements. More researches however are needed to address whether people should use iodine-containing supplements during pregnancy within the context of USI.

Our results showed that the iodine content in drinking water in participating counties was significantly lower than 100 μg/L, indicating that the environment in these areas are universally lacking iodine, according to the definition and demarcation of water-born iodine-excess areas and iodine-excess endemial areas (GB/T 19380–2016). Our results also showed that the iodine content in table salt, but not drinking water, was positively associated with UICs in participants. These results confirm that USI remains the main strategy to sustain elimination of IDD because the local environment is lacking iodine.

In this present study, we found that pregnant women living in the coastal areas had insufficient iodine intake, whereas those living in the inland ones were sufficient, which is consistent with other studies [35, 36]. Our study showed the coastal participants presented both a lower median UIC and a lower percentage of iodized salt than the inland participants. This regional difference by iodine status may be related with different household coverage of iodized salt between regions. Low consumption of iodized salt in the coastal regions may be explained as easy access along the coasts to raw sea salt without added iodine. Therefore, one approach may be to encourage pregnant women living in these areas to choose iodized salt over raw sea salt.

There are some limitations in our study. First, there are no data about dietary iodine sources. In addition to iodized salt, sea fish and seaweed are also sources of iodine. The recent studies in the coastal regions of China have revealed that iodized salt contributes approximately 60–80% of iodine requirement of local inhabitants [31, 37]. This figure demonstrates that iodized salt is the primary source of iodine. Second, there is no detailed information on the trimester of pregnancy among participants. Nevertheless, some studies with smaller samples of pregnant women in Zhejiang showed that there were no significant differences by trimester [18, 38]. Finally, this study was a clinic-based study with a relatively small sized sample. Thus, our results may not be applicable to all pregnant women in 90 counties across Zhejiang Province. Additional population-based, cross-sectional surveys are required.

## Conclusions

Our study showed that both household coverage of iodized salt and the consumption rate of adequately iodized salt were close to the targeted goats of Chinese National Standard of Elimination of IDD. However, pregnant women in Zhejiang overall were iodine deficient. Iodine intake status among pregnant women in this province showed regional differences. Pregnant women living in the coastal areas were mildly iodine- deficient whereas the inland participants were marginally sufficient. In addition to sustaining a USI strategy, more efforts (e.g. treatment with increased iodine content in salt or suggestion on iodine-containing supplements) are urgently needed to improve iodine intake during pregnancy, especially among pregnant women residing in the coastal regions.

## Abbreviations

CNIS 2011: The 2011 China’s National IDD Surveillance
IDD: Iodine deficiency disorder
IQR: Interquartile range
SE: Standard error
UIC: Urinary iodine concentration
USI: Universal salt iodization
USI: Universal salt iodization
WHO: World Health Organization

## References

[1] Zimmermann MB (2009). Iodine deficiency. Endocr Rev.

[2] Glinoer D (2007). The importance of iodine nutrition during pregnancy. Public Health Nutr.

[3] Yu CR, Wang YQ, Yao SR, Han YX, Chen YM, Zhu WM (1987). An epidemiological investigation on endemic goiter and endemic cretinism in Zhejiang province. Iodine deficiency disorders work group in Zhejiang provincial Center for Disease Control and Prevention, editors.

[4] Endemic Disease Control Department in Ministry of Public Health (1989). China National Programme 1989-1995 for the control of iodine deficiency disorders. Chin J Endemiol.

[5] Huang SM, Zhu WM, Yao SR, Zhou JS, Tu XG, Chen Y (2006). Analysis of surveillance on iodine deficiency disorders in Zhejiang province during 1995-2004. Chin J Endemiol.

[6] Zhou JS, Chen YM, Yao SR, Huang XM, Wang YQ, Tu XG (1999). Investigation and evaluation on the quality of iodine salt and the urine iodine level in the population of Zhejiang province. Zhongguo Di Fang Bing Xue Zazhi.

[7] Sun DJ, Codling K, Chang SY, Zhang SB, Shen HM, Su XH, et al. Eliminating iodine deficiency in China: achievements, challenges and global implications. Nutrients. 2017; 10.3390/nu9040361. 10.3390/nu9040361 PMC5409700 28379180

[8] Liu P, Su XH, Shen HM, Meng FG, Fan LJ, Liu SJ, Sun DJ, Xiao DL, Liu SJ (2014). A report on the 2011 China’s National Surveillance on IDD. The China’s national iodine deficiency diseases surveillance in 2011.

[9] Shan Z, Chen L, Lian X, Liu C, Shi B, Shi L (2016). Iodine status and prevalence of thyroid disorders after introduction of mandatory universal salt iodization for 16 years in China: a cross-sectional study in 10 cities. Thyroid.

[10] Fan LJ, Su XH, Shen HM, Liu P, Meng FG, Li M, Sun DJ, Lei ZL, Liu SJ (2014). A report on the 2011 China’s National Surveillance on IDD. The China’s National Surveillance Programme on IDD in 2014.

[11] Zimmermann MB, Andersson M (2012). Assessment of iodine nutrition in population: past, present, and future. Nutr Rev.

[12] World Health Organization. Assessment of iodine deficiency disorders and monitoring their elimination: A guide for programme managers (3rd ed). 2007. http://apps.who.int/iris/bitstream/handle/10665/43781/9789241595827_eng.pdf;jsessionid=315B2C879B526D132DC2022A2B8F4EA4?sequence=1. Accessed 15 Sept 2016.

[13] Mao GM, Ding GQ, Lou XM, Zhang RH, Zheng P, Mo Z (2015). Survey of iodine nutritional status in 2011, Zhejiang China. Asia Pac J Clin Nut.

[14] Zou Y, Lou XM, Ding GQ, Mo Z, Zhu WM, Mao GM. Iodine nutritional status after the implementation of the new iodized salt concentration standard in Zhejiang province China. BMC Public Health. 2014; 10.1186/1471-2458-14-836. 10.1186/1471-2458-14-836 PMC4139602 25118032

[15] Zou Y, Ding GQ, Lou XM, Mo Z, Zhu WM, Mao GM (2015). A study on the influencing factors of urinary iodine concentration and the relationship between iodized salt concentration and urinary iodine concentration. Br J Nutr.

[16] Zou Y, Lou XM, Ding GQ, Mo Z, Zhu WM, Mao GM, et al. A cross-sectional comparison study on the iodine nutritional status between rural and urban residents in Zhejiang province China. BMJ Open. 2014; 10.1136/bmjopen-2014-005484. 10.1136/bmjopen-2014-005484 PMC4078769 24969785

[17] Zou Y, Lou XM, Ding GQ, Mo Z, Zhu WM, Mao GM (2014). An assessment of iodine nutritional status and thyroid hormone levels in children aged 8-10 years living in Zhejiang province, China: a cross-sectional study. Eur J Pediatr.

[18] Mo Z, Lou XM, Zhu WM, Wang XF, Mao GM, Zhou JS (2013). A cross-sectional study on iodine nutrition in general population from Zhejiang province China. Chin J Endemiol.

[19] Zimmermann MB, Delange F (2004). Iodine deficiency of pregnant women in Europe: a review and recommendations. Eur J Clin Nutr.

[20] Bath SC, Rayman MP (2015). A review of iodine status of UK pregnant women and its implications for the offspring. Environ Geochem Health.

[21] Clifton VL, Hodyl NA, Fogarty PA, Torpy DJ, Roberts R, Nettelbeck T, et al. The impact of iodine supplementation and bread fortification on urinary iodine concentration in a mildly iodine deficient population of pregnant women in South Australia. Nutr J. 2013; 10.1186/1475-2891-12-32. 10.1186/1475-2891-12-32 PMC3621546 23497409

[22] Delshad H, Touhidi M, Abdollahi Z, Hedayati M, Salehi F, Azizi F (2016). Inadequate iodine nutrition of pregnant women in an an area of iodine sufficiency. J Endocrinol Investig.

[23] Simpong DL, Adu P, Bashiru R, Morna MT, Yeboah FA, Akakpo K, et al. Assessment of iodine status among pregnant women in a rural community in Ghana- a cross sectional study. Arch Public Health. 2016; 10.1186/s13690-016-0119-y. 10.1186/s13690-016-0119-y PMC4762158 26904197

[24] Versloot PM, Schroder-van JP, Heide D, Boogerd L (1997). Effects of marginal iodine deficiency during pregnancy: iodide uptake by the maternal and fetal thyroid. Am J Phys.

[25] Liu Y, Zhang L, Li J, Shan Z, Teng W (2013). Maternal marginal iodine efficiency affects the expression of relative proteins during brain development in rat offspring. J Endocrinol.

[26] Caron P (2015). Neurocognitive outcomes of children secondary to mild iodine deficiency in pregnant women. Ann Endocrinol.

[27] Hynes KL, Otahal P, Hay I, Burgess JR (2013). Mild iodine deficiency during pregnancy is associated with reduced educational outcomes in the offspring: 9-year follow-up of the gestational iodine cohort. J Clin Endocrinol Metab.

[28] Bath SC, Steer CD, Golding J, Emmett P, Rayman MP (2013). Effect of inadequate iodine status in UK pregnant women on cognitive outcomes in their children: results from the Avon longitudinal study of parents and children (ALSPAC). Lancet.

[29] Abel MH, Caspersen IH, Meltzer HM, Haugen M, Brandlistuen RE, Aase H (2017). Suboptimal maternal iodine intake is associated with impaired child neurodevelopment at 3 years of age in the Norwegian mother and child cohort study. J Nutr.

[30] Liu SJ, Su XH, Sun DJ, Shen HM, Zhang ST, Wei HL, Xiao DL, Sun DJ, Bai HQ, Liu SJ (2007). A report on the 2005 China’s National Surveillance on IDD. The China’s national iodine deficiency diseases surveillance in 2005.

[31] Wu Y, Li X, Chang S, Liu L, Zou S, Hipgrave DB (2012). Variable iodine intake persists in the context of universal salt iodization in China. J Nutr.

[32] Chen ZH, Xu LS, Wang MH, Wu JN, Meng HE, Min H (2011). Iodine nutritional status of residents in coastal areas of Fujian province. Chin J Endemiol..

[33] Wang MH, Chen ZH, Wu JN, Lan Y, Wu XY, Chen DQ (2016). Typical population iodine nutrition and health survey results analysis of Fujian province in 2014. Chin J Ctrl Endem Dis.

[34] Yang J, Zhu L, Li XF, Zheng HM, Wang Z, Liu Y (2016). Iodine status of vulnerable population in Henan province of China 2013-2014 after the implementation of the new iodized salt standard. Biol Trace Elem Res.

[35] Ghattas H, Francis S, EI Mallah C, Shatila D, Merhi K, Hlais S, et al. Lebanese children are iodine deficient and urinary sodium and fluoride excretion are positive predictors of urinary iodine. Eu J Nutr 2015:10.1007/s00394-015-1120-x. 10.1007/s00394-015-1120-x 26650194

[36] Ahad F, Ganie SA (2010). Iodine, iodine metabolism and iodine deficiency disorders revisited. Indian J Endocrinol Metab.

[37] Mao GM, Ding GQ, Huang LC, Lou XM, Zhang RH, Zhu WM (2013). Study on level of dietary iodine intake and its contribution rate of residents in Zhejiang. Chin J Prev Med.

[38] Lou XM, Mo Z, Ding GQ, Zhu WM, Mao GM, Zhou JS (2011). Surveys on iodine nutritional status of pregnant and lactating women in coastal areas of Zhejiang province. Chin J Endemiol.

